# Changes in the Mechanical Properties and Composition of Bone during Microdamage Repair

**DOI:** 10.1371/journal.pone.0108324

**Published:** 2014-10-14

**Authors:** Gang Wang, Xinhua Qu, Zhifeng Yu

**Affiliations:** 1 Shanghai Key Laboratory of Orthopaedic Implants, Department of Orthopaedic Surgery, Shanghai Ninth People's Hospital, Shanghai Jiaotong University School of Medicine, Shanghai, China; 2 Department of Orthopedic Surgery, the Second Affiliated Hospital of Nanjing Medical University, Nanjing, China; University of California Davis, United States of America

## Abstract

Under normal conditions, loading activities result in microdamage in the living skeleton, which is repaired by bone remodeling. However, microdamage accumulation can affect the mechanical properties of bone and increase the risk of fracture. This study aimed to determine the effect of microdamage on the mechanical properties and composition of bone. Fourteen male goats aged 28 months were used in the present study. Cortical bone screws were placed in the tibiae to induce microdamage around the implant. The goats were euthanized, and 3 bone segments with the screws in each goat were removed at 0 days, 21 days, 4 months, and 8 months after implantation. The bone segments were used for observing microdamage and bone remodeling, as well as nanoindentation and bone composition, separately. Two regions were measured: the first region (R1), located 1.5 mm from the interface between the screw hole and bone; and the second region (R2), located>1.5 mm from the bone-screw interface. Both diffuse and linear microdamage decreased significantly with increasing time after surgery, with the diffuse microdamage disappearing after 8 months. Thus, screw implantation results in increased bone remodeling either in the proximal or distal cortical bone, which repairs the microdamage. Moreover, bone hardness and elastic modulus decreased with microdamage repair, especially in the proximal bone tissue. Bone composition changed greatly during the production and repair of microdamage, especially for the C (Carbon) and Ca (Calcium) in the R1 region. In conclusion, the presence of mechanical microdamage accelerates bone remodeling either in the proximal or distal cortical bone. The bone hardness and elastic modulus decreased with microdamage repair, with the micromechanical properties being restored on complete repair of the microdamage. Changes in bone composition may contribute to changes in bone mechanical properties.

## Introduction

The skeleton is the main weight-bearing structure in humans. Under normal conditions, loading activities result in microdamage in the living skeleton, which is repaired by bone remodeling [1]. Microdamage is primarily caused by excessive continuous fatigue loading and mechanical loading, such as that generated by implantation [2]. Compared with the microdamage caused by fatigue loading, the microdamage resulting from implant surgery has been the focus of a limited number of studies, suggesting that the implantation procedure may cause extensive microdamage to the peri-implant bone [3]. Moreover, microdamage accumulation may impair the strength of the bone-implant interface, potentially causing implant loosening; compromise the mechanical properties of bone; and increase the risk of fracture [4].

Until recently, most studies have investigated the effect of prevalent microdamage on the mechanical properties of bone. To our knowledge, there is limited data regarding the restoration of bone quality after microdamage repair. Apparently, extensive microdamage often occurs in peri-implant bone, which is affected mainly by diffuse damage. Moreover, microdamage is removed by bone remodeling and—possibly—the deposition of mineralized tissue [5], [6], which may change the structure and mechanical properties of local bone. The present study aimed to determine the effect of microdamage on the mechanical properties and composition of bone. Overall, we were able to confirm our hypothesis that the accumulation and repair of microdamage changes the composition of local bone, which decreases the mechanical properties of bone.

## Materials and Methods

### Screw implantation and groups

**Figure 1:**
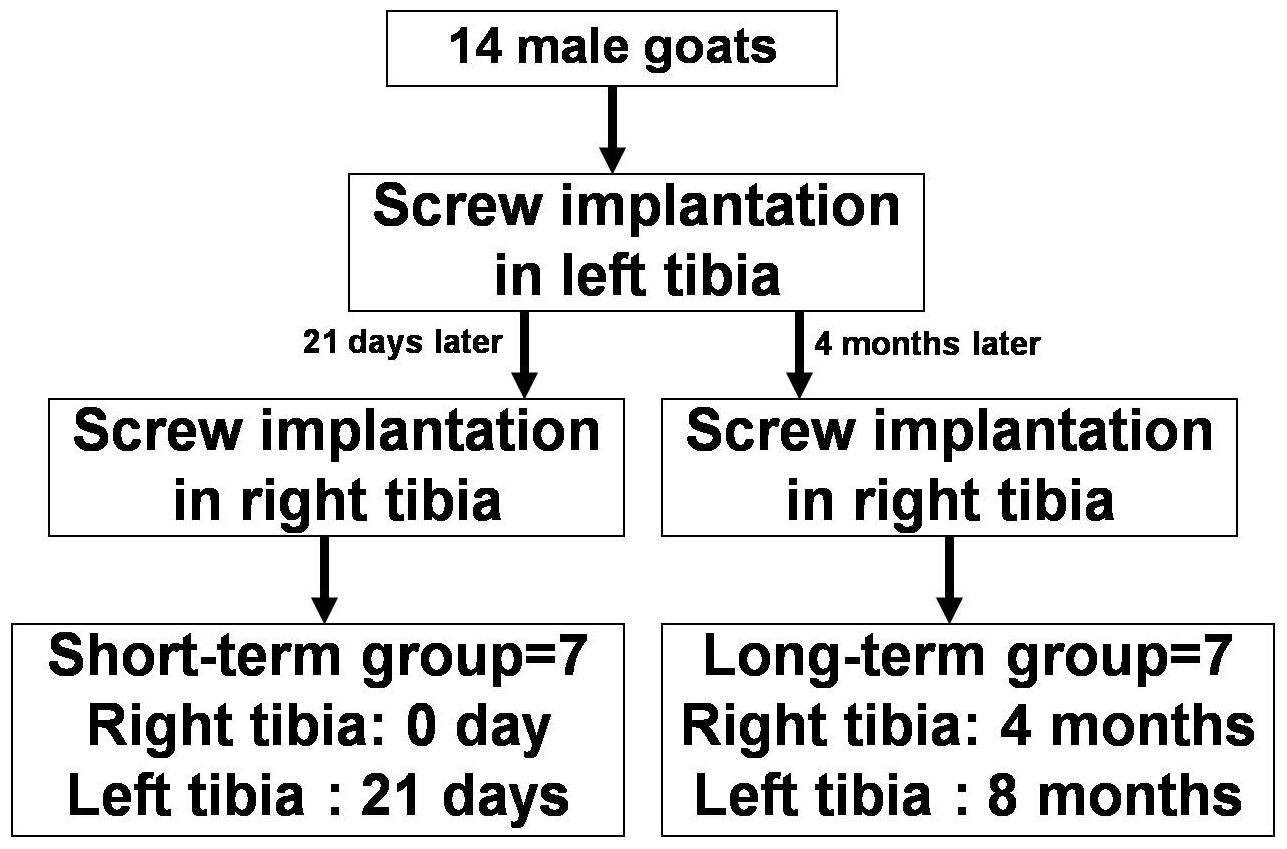
Schedule for screw implantation in goat tibiae in both groups.

Fourteen skeletally mature Chinese mountain goats with a body weight ranging from 27–32 kg were used in the present study. The goats are all male and aged 28 months. Skeletal maturity was confirmed by radiography showing closure of the distal femoral and proximal tibia growth plates [7]. The goats were kept on a farm and cared for by a qualified veterinarian during the entire study. Animal Research Ethics approval was obtained from the Research Ethics Committee of the Shanghai Ninth People's Hospital. Under pentobarbital sodium (50 mg/kg, IV) anesthesia, a longitudinal incision was made along the cranial-lateral aspect of the leg. In each tibia, 5 holes were drilled into the cranial-lateral diaphysis with the centered hole located at the midline of the tibia diaphysis. The screws only passed through one side of the cortex, and the distance between neighboring screws was 20 mm. After drilling and tapping, titanium cortical bone screws measuring 2 mm in diameter and 10 mm in length were inserted into each hole with a torque of 40 N·cm. The incisions were closed in layers using 4.0 vicryl sutures. All animals were injected with tetracycline (20 mg/kg, IV), 14 days before being euthanized, for 2 consecutive days. Seven days prior to necropsy, all animals were injected with calcein (15 mg/kg, IV), for 2 consecutive days. The schedules for screw implantation in each tibiae and the time of euthanasia are presented in Fig. 1. The bone segments with screws were removed and numbered according to the longitudinal direction. Segment 2 was stained with basic fuchsin, embedded in polymethyl methacrylate (PMMA) and cut cross-sectionally for the observation of microdamage and bone remodeling. Segment 4 was embedded in PMMA without basic fuchsin stain for nanoindentation testing. Segment 5 was used for bone composition testing. The samples taken at 0 days and 21 days were prepared for short-term observation, and the 4-month and 8-month samples were prepared for long-term observation. Fourteen goats were allocated to short-term (n = 7) and long-term (n = 7) observation separately (Fig. 1).

### Microdamage observation and histomorphometry measurements

**Figure 2:**
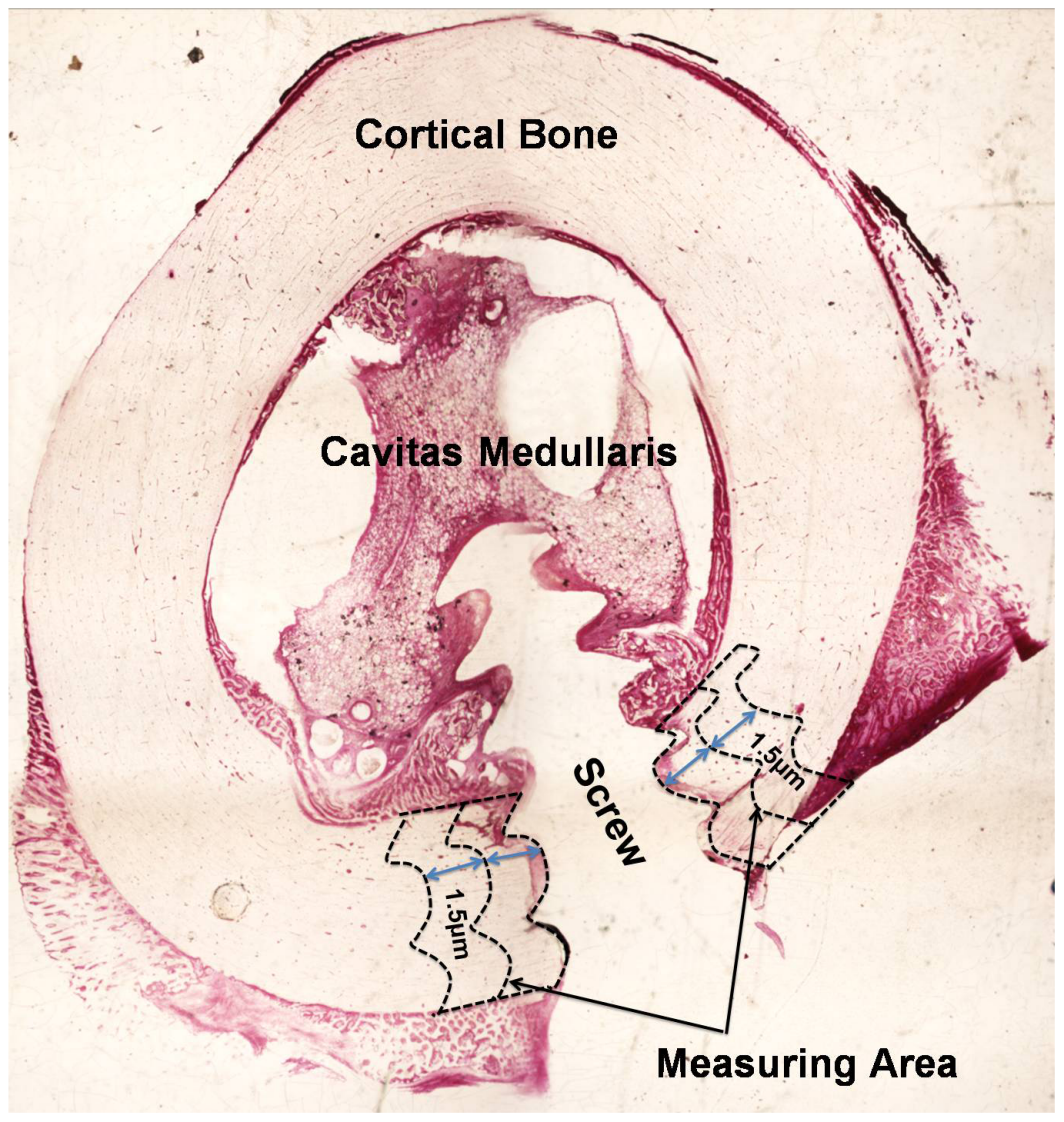
Schematic diagram of the measuring area.

After euthanasia, the bilateral tibiae of the goats were removed, and bone segments implanted with screws were prepared for bulk staining in basic fuchsin for microdamage analysis [8]. After basic fuchsin stained, screws were removed, bone samples of 2 cm in length were embedded in PMMA and cut into slices with a thickness of 50 µm using a hard tissue slicer (Leica SP1600, Germany). Stained sections were observed and measured under a fluorescence microscope. The Bioquant Image Analysis system (Bioquant OSTEO II VB.10.20, USA) was used to measure the porosity of the whole segments and the level of microdamage in the following 2 regions: the R1 region located 1.5 mm from the interface between the screw hole and the bone, and the R2 region located>1.5 mm (1.5∼3) from the bone-screw interface (Fig. 2) [9]. Diffuse damage often occurred in a narrow area adjacent to the bone-screw interface. In diffuse damage microcracks were too small to be discriminated by light microscopy. Therefore, pooled staining was present in bone with diffuse damage. In cross-hatched damage microcracks were vaguely distinguishable under fluorescence microscope [10]. The microdamage measurements included the damaged area in diffuse microdamage area (Dif.Dx.Ar = the area of diffuse microdamage/bone area) and the crack density (Cr.Dn = the number of cracks/bone area), mean crack length (Cr.Le), and crack surface density (Cr.S.Dn = the mean crack length/bone area) in the linear cracks [11]. The variables related to bone remodeling were measured in the same region of microdamage measurement. Moreover, the densities of the cortical porosity (Ct.Po = the number of resorption cavities/total bone area),mineralized surface (Md.S = the mineralized surface/bone surface) and mineral apposition rate (MAR = interlabel distance/label interval) were calculated.

### Nanoindentation measurement

The polished PMMA embedded bone blocks were examined using nanoindentation techniques as reported previously [12]. The elastic modulus (E) and contact hardness (H) were calculated using the Oliver-Pharr method [13], where the elastic modulus is a function of the unloading stiffness of the force-displacement curve, under the assumption that the unloading is elastic. Contact hardness is calculated as the maximum load divided by the projected area of the indenter tip at maximum load. A Nano Indenter XP system (MTS Nanoindenter XP, Oak Ridge, USA) was employed to measure the force and displacement during the indentation of the polished bone specimen. For each specimen, the sites selected for nanoindentation were consistent with the areas for microdamage measurement. Sixty-four points were selected per region. The measurement areas were determined using an optical microscope at 50× magnification. A Berkovich shape diamond indenter tip (Ei = 1141 Gpa, vi = 0.07) was used to perform the nanoindentation tests at each site. The indentation procedure was under displacement control. After the surface was identified, the indenter was advanced to 500 nm at a speed of 10 nm/s to avoid the effect of bone surface roughness. A typical indentation load-displacement curve includes a loading segment, a 10-s holding period at maximum load, an unloading segment, and a 50-s holding period for thermal drift measurement at 10% of maximum load.

### Bone composition measurement

JEOL JXA-8100 electron microprobe wavelength dispersive system (WDS) was employed to detect the kinds of elements contained in the bone segment by analyzing the wavelength (or energy) of characteristic X ray. Backscattered electron (BSE) images were produced by Oxford Instruments INCA energy dispersive system (EDS). Then through quantitative analysis, by comparing the diffraction intensity with standard sample, quantitative result can be got from the testing sample.

### Statistical analyses

All data have been tested by SPSS Statistics 18.0 (SPSS Inc., Chicago, USA) and demonstrated to have normal distributions with homogeneous variances. We used two-way analysis of variance to compare differences in microdamage, bone remodeling, nanoindentation, and bone composition between different time points in different regions. Differences between the R1 and R2 regions were analyzed by the paired sample *t*-test. P values of <0.05 were considered significant.

## Results

### Microdamage morphology around the implant

**Figure 3:**
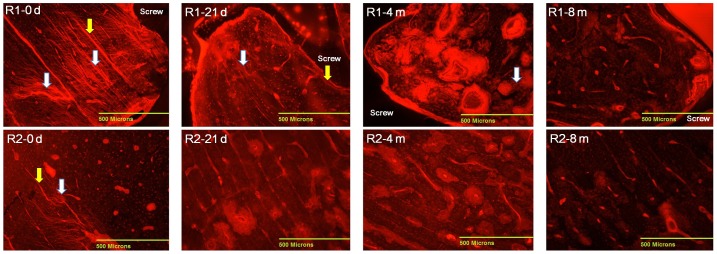
Microdamage accumulation and associated changes at 0 days, 21 days, 4 months, and 8 months after surgery (40×). White arrow referred to diffuse microdamage, yellow arrow referred to linear microcrack.

Under fluorescence microscopy, areas of microdamage dyed with basic fuchsin were observed around the implant in both regions. Diffuse microdamage and linear cracks were found in the cortical bone around the implants immediately after screw implantation (0 days), and diffuse microdamage was more easily observed than linear cracks in the R1 region (Fig. 3). Moreover, Fig. 3 shows that the diffuse microdamage and linear cracks were more pronounced in the R1 region.

### Region and time differences in the microdamage observed around the implant

**Figure 4:**
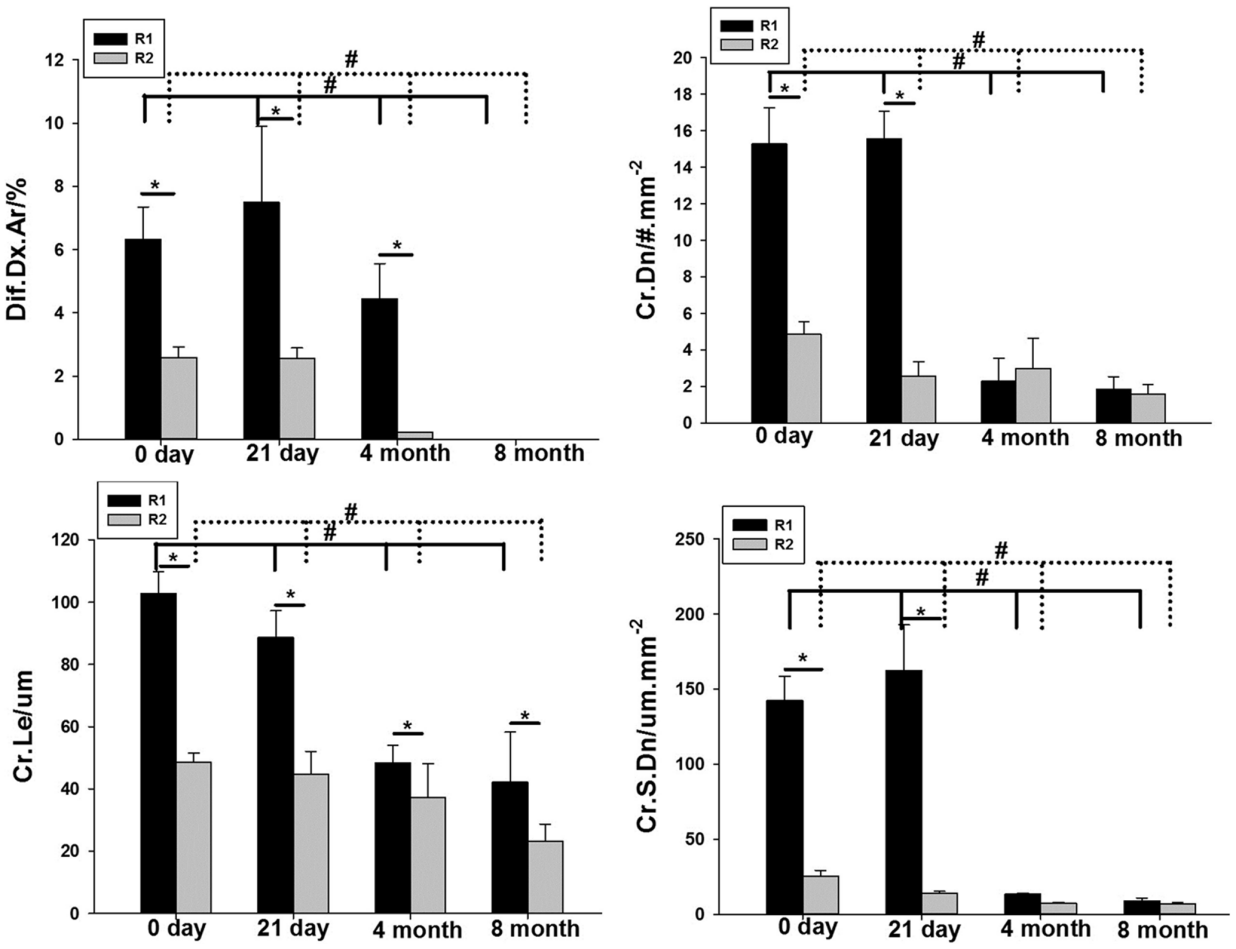
Production and repair of microdamage after screw implantation. * means difference between R1 and R2 region at different time point. # means difference between four time point in each region, solid line referred to R1 region, dotted line referred to R2 region.(*<0.0.5, #<0.05).

We used a two-way analysis of variance to determine the region and time differences between microdamage parameters. The Dif.Dx.Ar, Cr.Dn, Cr.Le, and Cr.S.Dn in the R1 region were significantly higher than those in the R2 region (Fig. 4), suggesting that screw implantation can produce more diffuse microdamage and more linear cracks around the screw. Interestingly, the values of the linear cracks, especially Cr.Dn and Cr.S.Dn, are relatively higher in the R1 region than the R2 region 21 days after surgery—5-fold and 10-fold higher in the R1 region, respectively—indicating that the linear cracks are more likely to be observed proximal to the screw.

### Repair mode for microdamage

**Figure 5:**
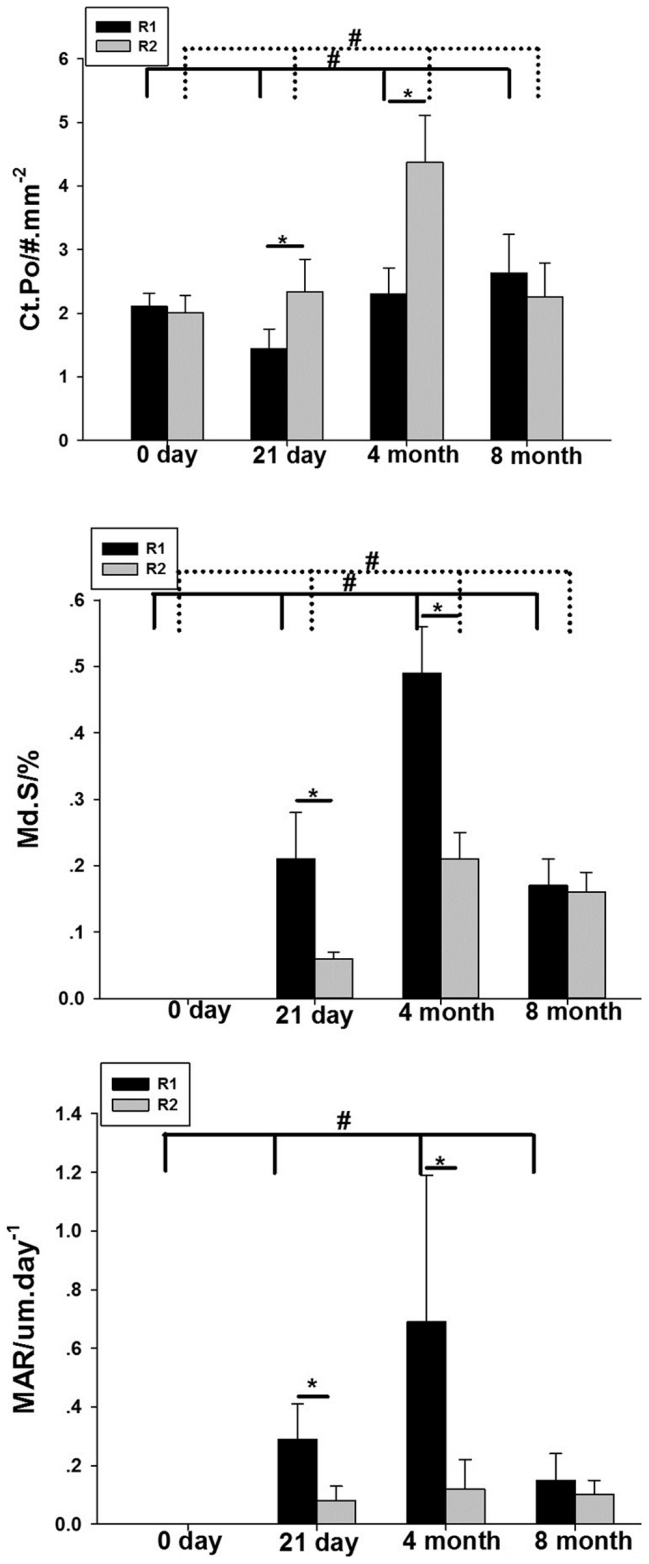
Comparison of bone remodeling parameters in different regions at different time points. * means difference between R1 and R2 region at different time point. # means difference between four time point in each region, solid line referred to R1 region, dotted line referred to R2 region.(*<0.0.5, #<0.05).

By comparing the results between different regions, we found no significant difference between the different regions at 0 days. At 21 days and 4 months, the labeled surface and bone porosity areas were significantly higher in the R1 than in the R2 region. However, at 8 months, although the bone porosity was higher in the R1 region, the labeled surface area was not different from that in the R2 region. Mineral apposition rate (MAR) changed greatly after surgery, especially in the R1 region. MAR in R1 region is higher than it in the R2 region after 21 days and 4 months of surgery. (Fig. 5).

### Change in micromechanical properties

**Figure 6:**
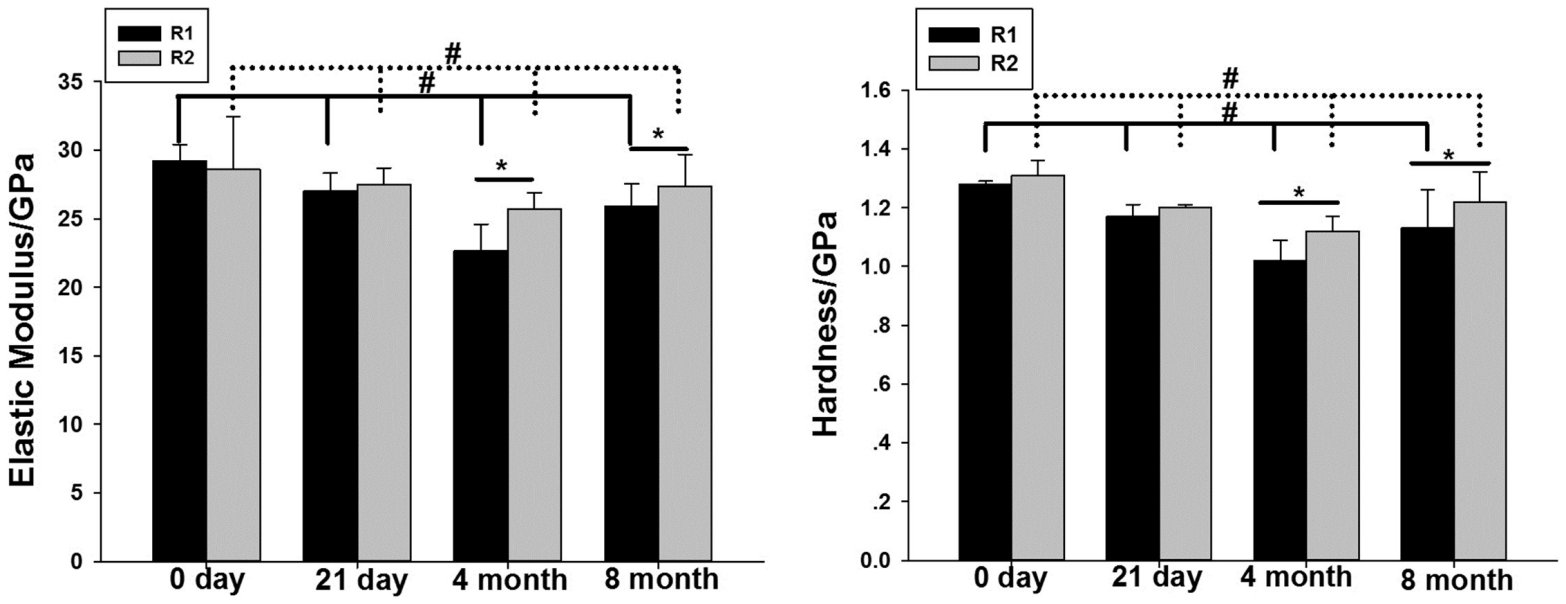
Comparison of nanoindentation data in different regions at different time points. * means difference between R1 and R2 region at different time point. # means difference between four time point in each region, solid line referred to R1 region, dotted line referred to R2 region.(*<0.0.5, #<0.05).

Nanoindentation testing showed that at different time points, the hardness and elastic modulus from 0 days, 21 days to 4 months showed a downward trend in both regions. Both of the hardness and elastic modulus began to increase after 4 month in R1 and R2 region. The hardness and elastic modulus of the bone tissue adjacent to the screw thread was significantly lower in the R1 than in the R2 region 4 months after surgery. After 8 months, the bone quality recovered slightly (Fig. 6). There was significant time difference among the 4 time points in each group (P<0.01).

### Change in bone composition

**Figure 7:**
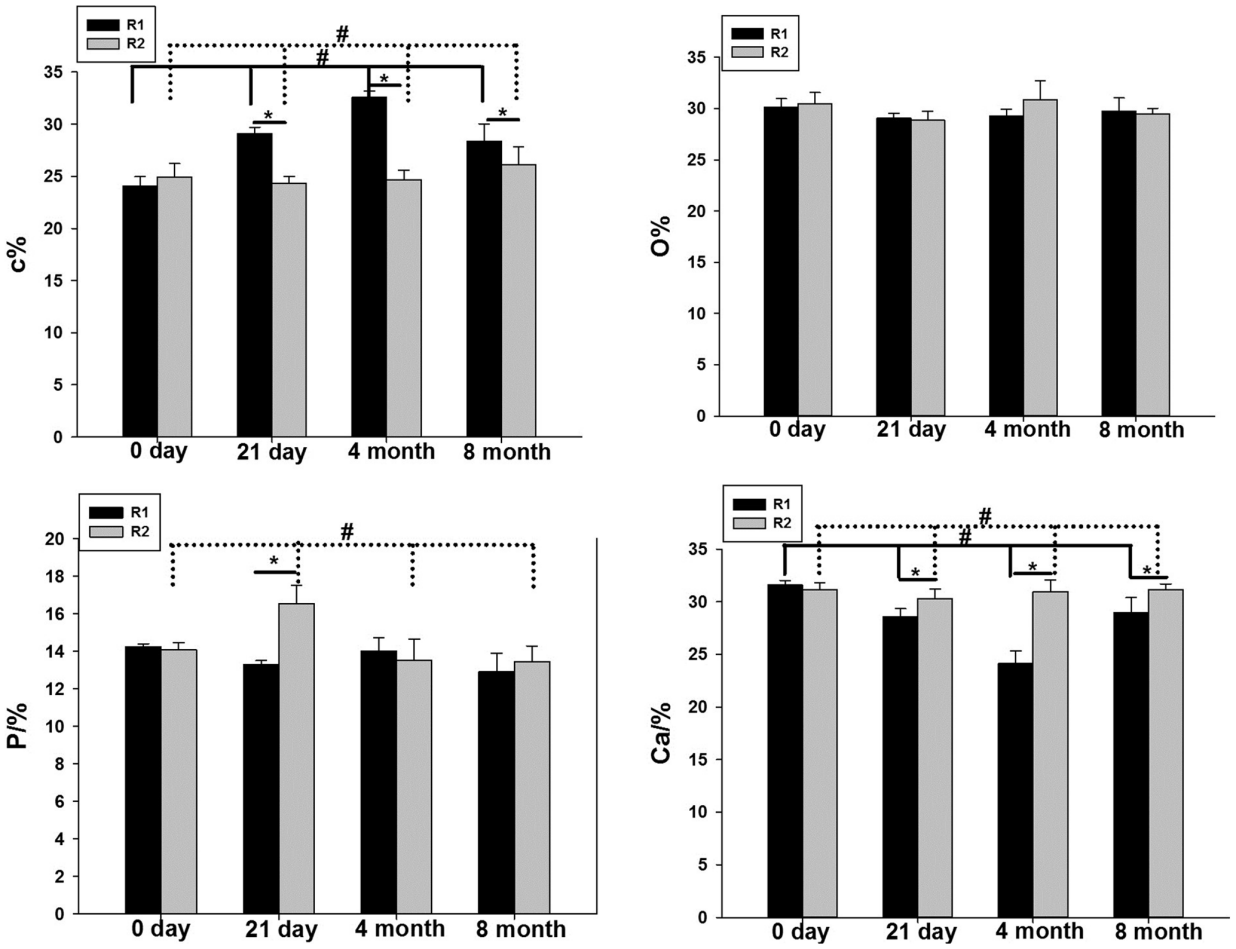
Comparison of bone composition (C, O, P, and Ca) in different regions at different time points. * means difference between R1 and R2 region at different time point. # means difference between four time point in each region, solid line referred to R1 region, dotted line referred to R2 region. (*<0.0.5, #<0.05).

We measured 4 elements, namely, C (Carbon), O (Oxygen), P (Phosphorus), and Ca (Calcium), in different regions. There was no difference between the 4 elements 0 days after surgery (P>0.05). At 21 days, significant differences were observed in the C, P and Ca elements between the R1 and R2 regions (P<0.05), with C being higher in the R1 region, and P and Ca being higher in the R2 region. At 4 months, C increased to peak levels in the R1 region, whereas Ca decreased to a minimum. At 4 months and 8 months, the C and Ca composition was significantly different between the R1 and R2 regions (P<0.01), whereas the O and P composition was not significantly different between groups (P<0.05, Fig. 7).

## Discussion

Although microdamage accumulation and increased bone remodeling are clearly evidenced in peri-screw bone [10], [14], the mechanisms of microdamage repair around the implant and change in the mechanical quality and composition of bone after screw insertion remain unclear. Previous studies suggest that the activation of bone resorption is considerably less effective in diffuse than in linear damage, suggesting that the response to diffuse and linear damage is different [15], [16]. However, the large-size linear cracks are known to cause severe damage to the bone matrix, leading to osteocyte apoptosis, which initiates bone remodeling [16] and acts as the primary factor contributing to targeted bone remodeling [16]. Overall, the mechanical properties of bone and mode of microdamage repair seem to depend on the different types of microdamage.

With regard to the location of the microdamage, Wang et al. [10] reported that diffuse damage tended to appear in the area adjacent to the bone-screw interface, which seems unlikely. In the present study, we frequently observed a combination of diffuse damage and linear cracks in peri-screw bone, which was referred to as complex microdamage. However, the diffuse microdamage decreased as the distance to the implant increased, and the linear cracks were still observed. As confirmed by O'Brien et al. [17], implant surgery on the cortical bone results in increased microdamage produced near the implant, owing to the greater damage to the proximal bone. Moreover, the complex microdamage is likely to be caused by the strong impact—the drilling and cutting forces in implant surgery result in rapid damage to bone. After surgery, implant micromotion is likely to increase microdamage accumulation.

Microdamage has been postulated as a contributing factor for bone remodeling, which replaces the damaged bone with new bone. Thus, the rate of bone remodeling increased in the area with microdamage, with Wang et al. [10] which suggested that bone resorption becomes considerably active within 1 month after screw implantation. Moreover, increased bone resorption was observed which may decreased diffuse damage and linear cracks. Although screw implantation can result in different types of microdamage in cortical bone, the type of microdamage associated with activation of bone remodeling remains unclear. A previous study by Bentolila et al. [15] reported that intracortical resorption was preferentially associated with linear-type cracks, with a 40% reduction in linear microcracks occurring 10 days after fatigue loading. In contrast, diffuse microdamage did not show a significant decrease at that time. In our study, we found that the bone resorption cavity and mineralized surface increased to peak levels 4 months after surgery, while the linear crack decreased rapidly compared with that 21 days after surgery, suggesting that linear microdamage is the main contributor to activation of bone remodeling. By using bisphosphonate coating screw, Agholme et al [18] found bisphosphonate coating leads to increased bone volume around screws. This maybe caused by preventing resorption of the tissue with microdamage and provide a new way to increase implant stability. But how much bisphosphonate were released to the bone and the change of bone remodeling should be confirmed, because bisphosphonate could increases microdamage accumulation during early treatment [19].

Moreover, the accumulation of microdamage has been associated with a reduction in the elastic modulus and strength of bone tissue, and an increase in energy dissipation when the bone is loaded [1], [4], [17], [20]–[22]. Although the effect of diffuse damage on bone strength remains unclear [17], the accumulation of microcracks at lower stress levels is believed increase the strength of cortical bone [23]. In fact, extensive microdamage often occurs in peri-implant bone, which is affected mainly by diffuse damage. Moreover, microdamage is removed by bone remodeling and—possibly—the deposition of mineralized tissue [5], [6], which may both change the structure and mechanical properties of local bone. In the early stage of repair, multiple resorption cavities impair bone structure and act as stress concentrators, both of which reduce the mechanical properties of bone [6]. During that period, the ideal osteointegration of the implant has not been established. Our results suggest that the hardness and elastic modulus did not change significantly during the early stage. However, after 4 months, with an increase in bone remolding and decrease of microdamage, the mechanical properties of local bone began to decrease. The area located near the implant—where significant microdamage occurred—was characterized by a greater decrease in the mechanical properties of bone than the region located far away from the implant. Both of elastic modulus and hardness declined until 4 months after surgery. At the same time, bone remodeling results show that they increased significantly after surgery. The decreased bone mechanical property may be caused by newly formed bone tissue being present following resorption.

The material properties of bone are determined by the matrix composition, including minerals, collagen and water—each of which can influence the mechanical properties of bone [24]. Micropetrotic bone is characterized by the loss of osteocytes, decreased water content and high mineralization [5], [25], [26]. However, bone remodeling—although increased—is not sufficient to rapidly repair all microdamage, particularly for extensive diffuse damage. The coexistence of lower mineralized new osteonal bone and higher mineralized micropetrotic bone would further compromise the mechanical properties of bone. Accordingly, the bone quality around the endosseous implant may not return to normal immediately after microdamage repair. Moreover, increased diffuse damage may change the normal repair mode, which may compromise the restoration of bone quality after microdamage repair. By using BSE [27] and Fourier transform infrared (FTIR) [28] imaging, previous studies have found that calcium and collagen contribute to the mechanical properties of bone. In the present study, although we did not measure changes in collagen, we did measure changes in C, O, P, and Ca. We found that 4 elements—especially C and Ca—changed greatly in the region surrounding the screw during the production and repair of microdamage. The C levels were highest 4 months after surgery, while the Ca levels were lowest at that time. Moreover, the above results suggest that bone remodeling increased to a maximum 4 months after surgery. The variation trend of bone composition may caused by bone remodeling during repair of microdamge. Change of Ca was the same as elastic modulus and hardness, but because the sample size is too low, we cannot find strong correlation between Ca and bone mechanical property.

Overall, both diffuse microdamage and linear cracks were found around the implant and accumulated during the early stage after surgery, and these types of damage may impair the initial stability of the implant. The decreased microdamage at later stages after surgery indicates that the implant-induced microdamage was repaired.The repair of microdamage by bone remodeling can change the structure and mechanical properties of local bone. Accordingly, the bone will return to normal after microdamage repair, through further remodeling activated by mechanical stimulation or other factors, which may take a relatively long time.
