# Correction: Changes in the Mechanical Properties and Composition of Bone during Microdamage Repair

**DOI:** 10.1371/journal.pone.0216435

**Published:** 2019-04-30

**Authors:** Gang Wang, Xinhua Qu, Zhifeng Yu

In Fig 3, the representative image of microdamage staining of the R1 region is incorrect. The authors have provided a corrected version here.

**Fig 3:**
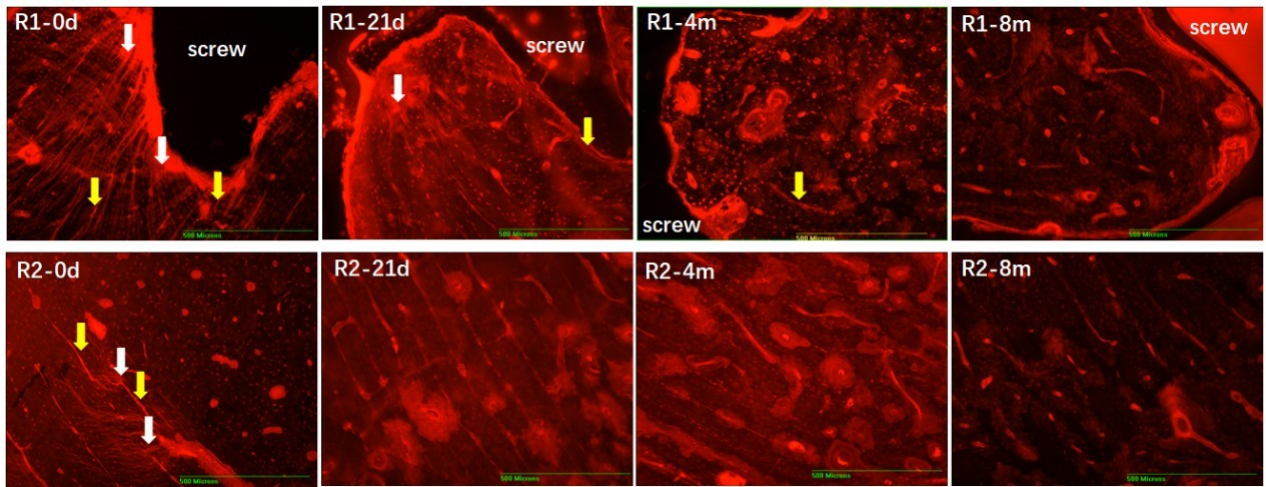
Microdamage accumulation and associated changes at 0 days, 21 days, 4 months, and 8 months after surgery (40×). White arrow referred to diffuse microdamage, yellow arrow referred to linear microcrack.

## References

[1] Wang G, Qu X, Yu Z (2014) Changes in the Mechanical Properties and Composition of Bone during Microdamage Repair. PLoS ONE 9(10): e108324 10.1371/journal.pone.0108324 25313565 PMC4196754

